# Supplementary material for: Integrated Metabolomics and Transcriptomics Provide Key Molecular Insights into Floral Stage-Driven Flavonoid Pathway in Safflower
Source: Int J Mol Sci. 2024 Nov 6;25(22):11903. doi: 10.3390/ijms252211903 (PMC11593580; doi:10.3390/ijms252211903)

Figure S1：Qualitative and quantitative metabolite analysis

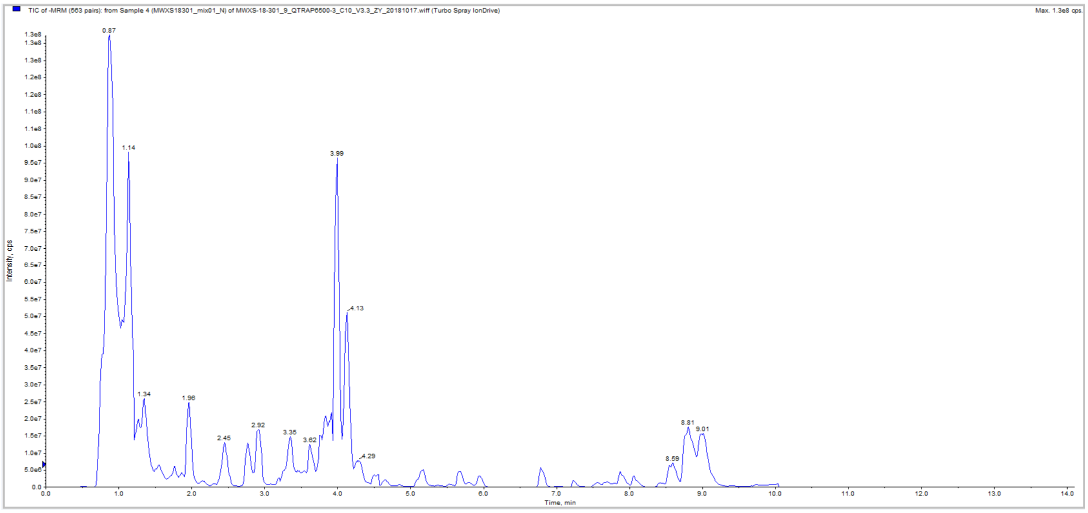

a: Mixed-sample mass spectrometry analysis of total ion flow plots

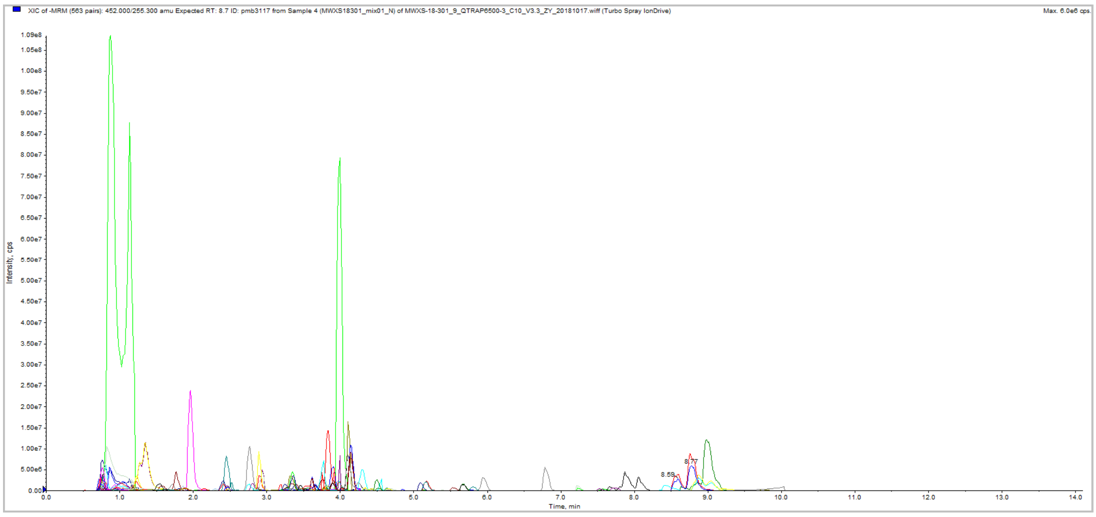

b: MRM Metabolite Assay Multi-Peak Plot

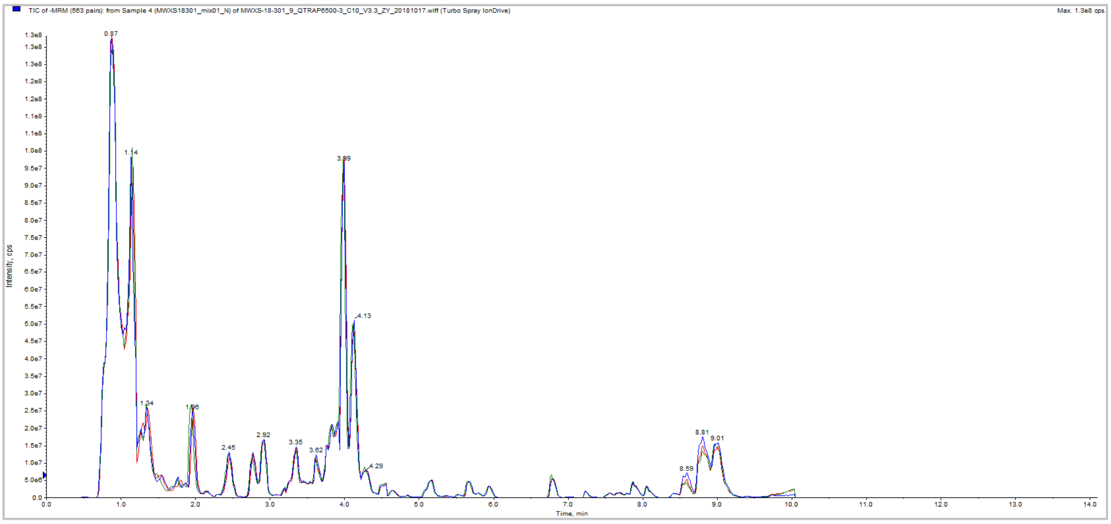

c: Overlay of TIC for mass spectrometry detection of QC samples

Figure S2：GO enrichment analysis of DEGs between three groups

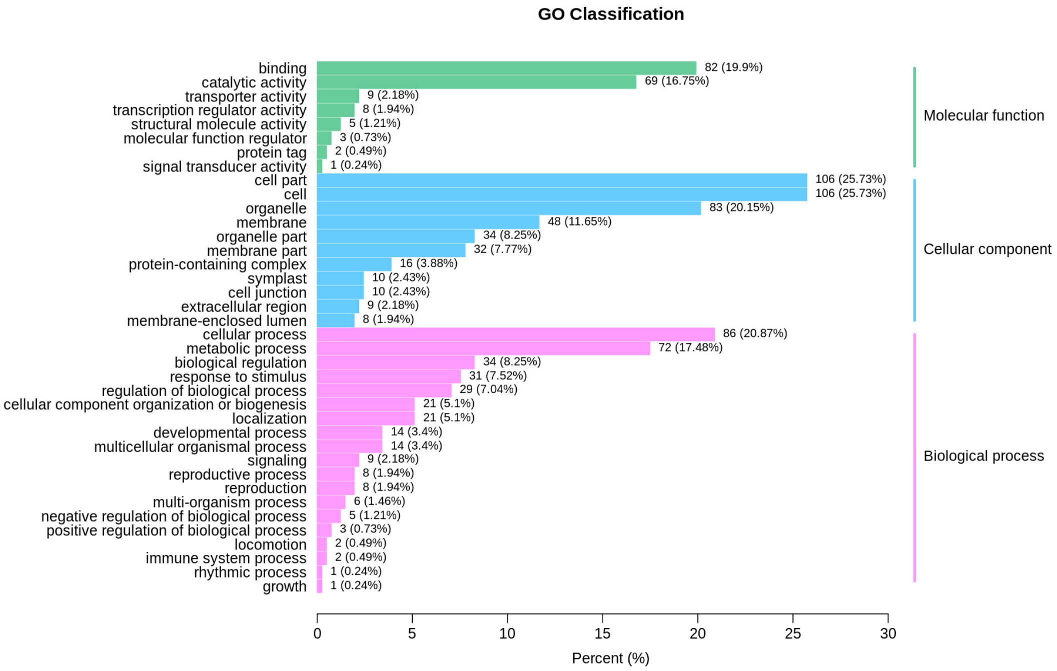

a: GO enrichment analysis of DEGs between INvsFU

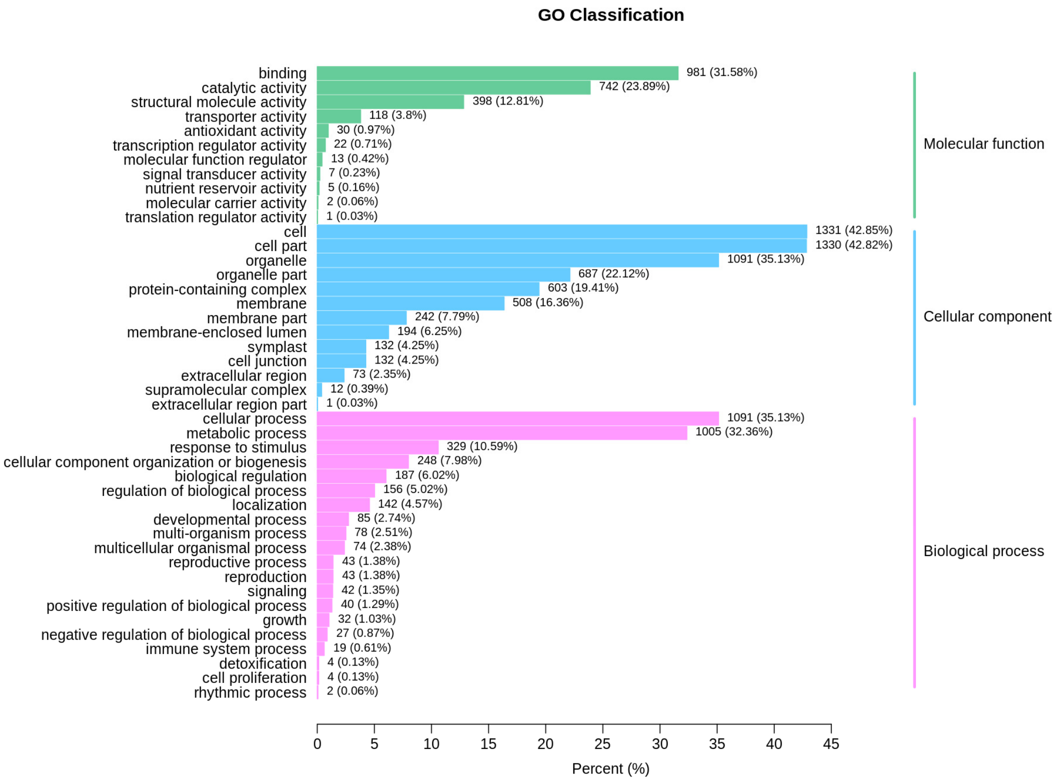

b: GO enrichment analysis of DEGs between INvsFA

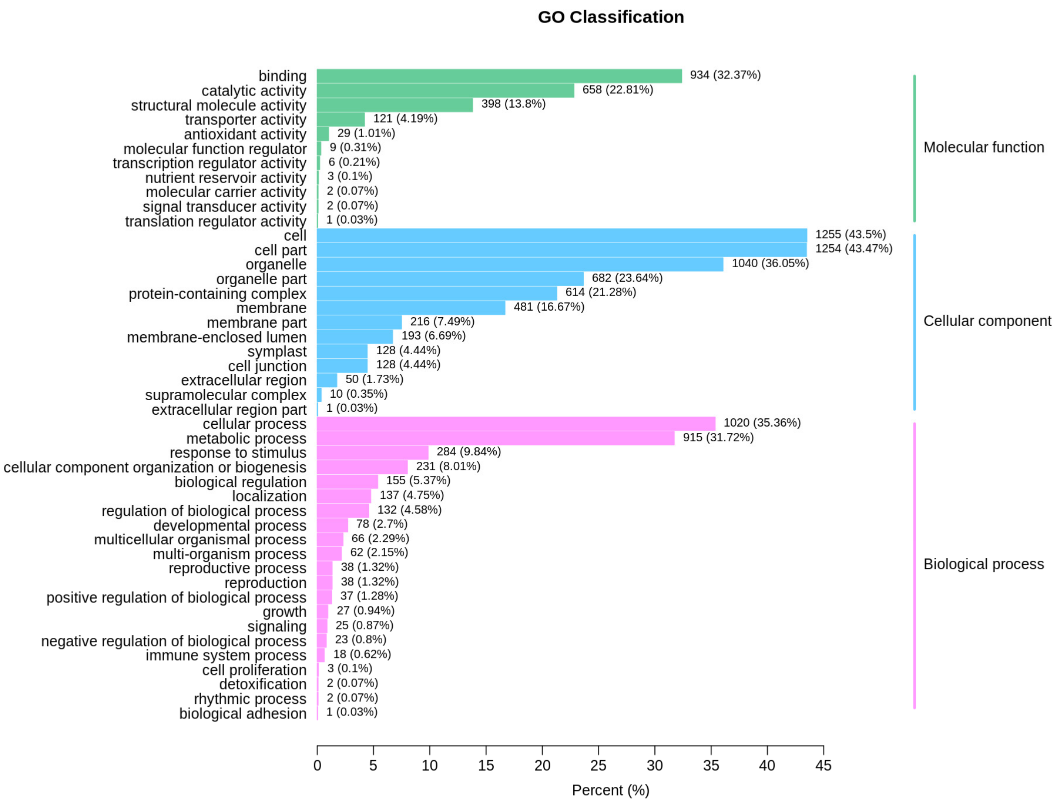

c: GO enrichment analysis of DEGs between FUvsFA

Figure S3: Three periods of petals

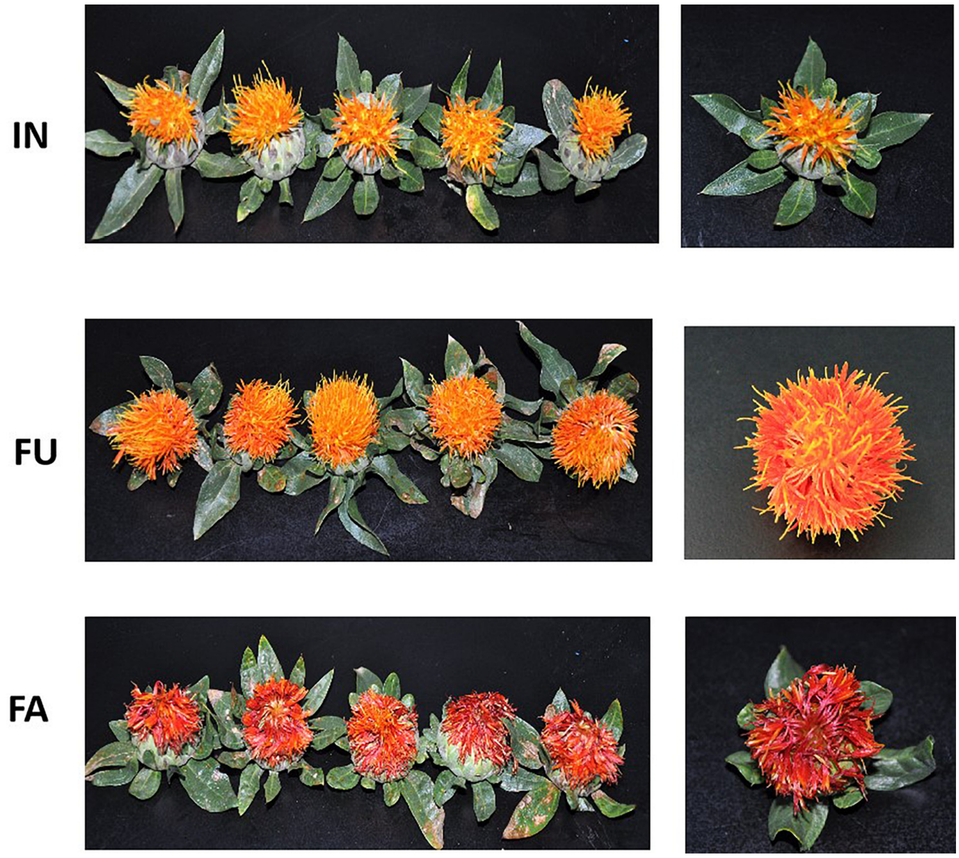

Supplement: Supplementary file 1 [file ijms-25-11903-s001.zip › Supplementary Figures.pdf]
